# Variation of Soil Microbial Community and Sterilization to *Fusarium oxysporum* f. sp. *niveum* Play Roles in Slightly Acidic Electrolyzed Water-Alleviated Watermelon Continuous Cropping Obstacle

**DOI:** 10.3389/fmicb.2022.837121

**Published:** 2022-04-28

**Authors:** Xue Wu, Cuinan Wu, Daipeng Lu, Yiwen Wu, Zhangying Ye, Liru Xia, Yudong Sun, Encai Bao, Lin Ye, Yuxin Tang, Kai Cao

**Affiliations:** ^1^The Agriculture Ministry Key Laboratory of Agricultural Engineering in the Middle and Lower Reaches of Yangtze River, Institute of Agricultural Facilities and Equipment, Jiangsu Academy of Agricultural Sciences, Nanjing, China; ^2^School of Biosystems Engineering and Food Science, Zhejiang University, Hangzhou, China; ^3^School of Agricultural Engineering, Jiangsu University, Zhenjiang, China; ^4^Jiangsu Xuhuai Region Huaiyin Institute of Agricultural Science, Huai’an, China; ^5^Agriculture College, Ningxia University, Yingchuan, China

**Keywords:** soil microbial diversity, *Fusarium oxysporum* f. sp. *niveum*, watermelon, continuous cropping obstacle, slightly acidic electrolyzed water

## Abstract

It is critical to exploit technologies for alleviating watermelon continuous cropping obstacle which frequently occurs and results in the limiting production and economic losses of watermelon. This study aimed to explore the effects of slightly acidic electrolyzed water (SAEW) on watermelon continuous cropping obstacles. The results showed that SAEW significantly improved the growth of watermelon seedlings cultivated in continuous cropping soil and caused a mass of changes to the diversity of the soil microbial community. Compared with Con, SAEW decreased the diversity index of bacteria by 2%, 0.48%, and 3.16%, while it increased the diversity index of fungus by 5.68%, 10.78%, and 7.54% in Shannon, Chao1, and ACE index, respectively. Besides, the enrichment level of *Fusarium oxysporum* f. sp. *niveum* (FON) was remarkably downregulated by 50.2% at 14 days of SAEW treatment, which could decrease the incidence of *Fusarium* wilt disease. The wet and dry weights of FON mycelia in the fluid medium were depressed more than 93%, and the number of FON colonies in continuous cropping soil was reduced by 83.56% with SAEW treatment. Additionally, a strong correlation between watermelon, FON, and SAEW was presented by correlation analysis. Furthermore, the content of endogenous reactive oxygen species (ROS) was over quadruply increased by SAEW, which may contribute to the sterilizing effect of SAEW on FON. Taken together, our findings demonstrated that exogenous SAEW could alter the soil microbial diversity and decrease the accumulation of FON, which improved the growth of watermelon seedlings and finally alleviated continuous cropping obstacles of watermelon.

## Introduction

Watermelon (*Citrullus lanatus*) is an important cucurbitaceous and globally cultivated cash crop in China, Iran, and Turkey, and its production is already over 119 million tons per year in 2017 (Guo et al., 2013; FAOSTAT, 2017; Wang et al., 2019). However, continuous cropping obstacle has limited watermelon production in many areas around the world and resulted in an important economic loss of watermelon (An et al., 2011). Continuous cropping, to wit the cultivation of the same variety of crop or its related varieties on the same cropland for a long term, could lead to the continuous cropping obstacle. Continuous cropping could change the chemical properties and microbial communities of soil, as well as increase the possibility of plant autotoxins and soil-borne diseases (Ling et al., 2014; Chen et al., 2020; Liu et al., 2020; Zhang et al., 2020). Moreover, it has been proven that *Fusarium* wilt induced by *Fusarium oxysporum* f. sp. *niveum* (FON) is a serious soil-borne disease raised from continuous cropping obstacle in various crops including tobacco, strawberry, cotton, peanut, and watermelon (Li H. et al., 2019; Wang et al., 2019; Li et al., 2020; Ding et al., 2021). Therefore, it is critical to repair the rhizosphere environment in the soil to control the menace induced by FON.

Until now, a number of studies have shown that continuous cropping gave rise to the disruption of soil microbial community composition (Li et al., 2014; Qin et al., 2017; Yang et al., 2017; Wang et al., 2019; Li et al., 2020). For instance, the bacterial and fungal diversities were observably altered after the long-term monoculture of banana and watermelon (Shen et al., 2018; Wang et al., 2019; Ding et al., 2021). That is, clarifying the effect on soil microbial community was a key indicator to assess the availability of ameliorative measures in alleviating continuous cropping obstacles. Recently, different methods have been used to solve the problem of watermelon continuous cropping obstacle, including soil sterilization, crop rotation, grafting technique, and use of compost (Xu et al., 2015; Lv et al., 2018; Li C. et al., 2019; Li et al., 2020; Toledo et al., 2020; Ding et al., 2021; McDonald et al., 2021). However, some of the traditional ameliorative strategies usually take a long time to work or even have the possibility of damages to the environment and changes in watermelon quality. Therefore, it is essential to screen out the safe, convenient, and environment-friendly measures to ameliorate the resistance of watermelon to continuous cropping obstacles.

Slightly acidic electrolyzed water (SAEW) is a late-model disinfectant produced by the electrolytic dilute hydrochloric acid or sodium chloride solutions or both in an electrolytic bath. SAEW is colorless, odorless, less corrosive to human, equipment, and friendly to the environment with its subacid pH (5.0-6.5). It has been directly applied to extend the shelf-life of food in America and Japan as well as used in the industry of fruits and vegetables due to its great bactericidal efficacy with a minimal harm to the physiochemical properties of agricultural products (Hao J. et al., 2011; Hao et al., 2021; Ding et al., 2015, 2016; Xuan et al., 2016). The well bactericidal effect of SAEW was on account of the available chlorine compounds, including ClO-, HClO, and Cl_2_ (Cao et al., 2009; Ye et al., 2017). Reactive oxygen species (ROS) is known to generate cell death in various types of cells (Dimmeler and Zeiher, 2000; Ye et al., 2017). Furthermore, a previous study has shown that SAEW could sterilize *Escherichia coli* by inducing abundant ROS to accumulate in cells (Ye et al., 2017). However, few studies were relevant to the application of SAEW in the environmental amelioration of crop cultivation and sterilization of FON. In this study, the effects of SAEW on the growth of watermelon seedlings cultivated in continuous cropping soil, the microbial diversity of soil, and the bacteriostasis to FON by accumulating excess ROS were investigated. Our findings helped to figure out the underlying mechanism of ameliorating continuous cropping obstacles by SAEW and further revealed the application prospect of SAEW in watermelon cultivation.

## Materials and Methods

### Experimental Design

Continuous cropping soil was collected from a field that was continuously planted with watermelon for more than 7 years from the Ningxia Hui Autonomous Region of China (36°59′N, 105°15′E, with an average altitude of 1,723 m). The soil sampling point is shown in Supplementary Figure 1. Approximately 15-20% of the soil-borne disease incidence rate occurred in the soil sampling field, and the watermelon production of this field was approximately 17,710 kg⋅hm^–2^. The physical and chemical properties of sampling soil (0-20 cm soil layer) were described as follows: the test soil belonged to light gray calcium soil with the texture of sandy loam. The total nitrogen, phosphorus, potassium, and organic matter content were 1.0 g/kg, 6.2 mg/kg, 88 mg/kg, and 6.39 g/kg, respectively. The soil bulk density was 1.42 g/cm^3^ with 27.7% field water capacity. The experimental watermelon variety was “Jincheng NO.5.” Five treatment groups were established in this experiment, named Con (control, deionized water), SAEW20 (20 ppm concentration of SAEW), SAEW40 (40 ppm concentration of SAEW), SAEW60 (60 ppm concentration of SAEW), and SAEW80 (80 ppm concentration of SAEW). Watermelon seeds were germinated at 30°C for 48-72 h. The following growth to two true leaves and a terminal bud, uniform watermelon plants were transplanted in plastic flowerpots (12 cm in diameter and 12 cm in height) filled with soil samples. The Hoagland’s nutrient solution was used to fulfill the nutritional requirements of the watermelon seedlings. SAEW was applied every 3 days, and the growth index was measured after the plants grew to four true leaves and a terminal bud. A total of 15 pots were used in each treatment, and growth indices were measured with three independent experiments (*n* = 45).

### Preparation of Slightly Acidic Electrolyzed Water

Slightly acidic electrolyzed water was produced by electrolysis of 6% HCl solution using a generator (HD-240L, Fuqiang-Want Sanitary Accessories Ltd., Shanghai, China) with a non-membrane electrolytic chamber at a voltage of 220 V. The components of SAEW were ClO^–^ and HClO. SAEW was prepared when it was going to be used. Then, the pH and the available chlorine concentration of SAEW were determined by a pH meter (FE-28, METTLER TOLEDO, Shanghai, China) and chlorine concentration test paper (Newstar paper, Q/HSSC 202-2016, Hangzhou, China), respectively.

### Soil Sampling, DNA Extraction, and Sequencing

The effect of SAEW on the soil microbial community structure was accomplished *via* high-throughput sequencing. The soils with different treatments (Con, Water-7, Water-14, SAEW-7, and SAEW-14) were separately collected from 5 replicated pots for each. There was one watermelon seedling cultivated in every pot for each treatment. Con represented the original continuous cropping soil dug from the cultivated land of watermelon continuous monoculture in Ningxia Hui Autonomous Region of China. Water-7 and Water-14 represented the continuous cropping soil irrigated with deionized water for 7 and 14 days, respectively. SAEW-7 and SAEW-14 severally represented the continuous cropping soil irrigated with SAEW for 7 and 14 days. For each sample, 5 random soil cores (0–10 cm in depth) from each pot were collected as one soil sample. The 25 soil samples were separately placed into aseptic test tubes and stored at −80°C for subsequent DNA extraction. The total DNA of the microbial genome for each sample was extracted using the DNeasy PowerSoil Kit, according to the operation manual. A NanoDrop ND-1000 spectrophotometer and agarose gel electrophoresis were used to verify the quantity and quality of DNA. The V3–V4 region of bacterial 16S rRNA genes was amplificated by PCR (95°C for 2 min, followed by 35 cycles of 95°C for 30 s, 60°C for 45 s, and 72°C for 90 s, with a final extension of 72°C for 10 min) in triplicate 50 μl reaction volume by TransGen High-Fidelity PCR SuperMix (TransGen Biotech, Beijing, China) with the forward primer 341F (5′- CCTACGGGNGGCWGCAG-3′) and the reverse primer 806R (5′-GGACTACHVGGGTATCTAAT-3′) (Wang et al., 2020; Ding et al., 2021). Nuclear ribosomal internal transcribed spacer 1 (ITS1) regions were amplified by the forward primer ITS5F (5′- CTTGGTCATTTAGAGGAAGTAA-3′) and the reverse primer ITS2 (5′- GCTGCGTTCTTCATCGATGC-3′) in the same PCR protocol as 16S rRNA genes (Wei et al., 2020). Purified amplicons were pooled in equimolar, and the paired-end sequencing (PE250) was constructed on an Illumina platform of Guangzhou Gene *Denovo* Biotechnology Co., Ltd. according to the protocols. The DNA products of each soil sample were reacted three times. Then, the raw sequence reads were quality filtered by FASTP (version 0.18.0) as described by Chen et al. (2018). The paired-end clean reads were merged as raw tags using FLSAH (version 1.2.11) with a minimum overlap of 10 bp and mismatch error rates of 2%, which were shown in Magoè and Salzberg, 2011. The clean tags were clustered into operational taxonomic units (OTUs) of ≥ 97% similarity using UPARSE (version 9.2.64) pipeline as described by Edgar, 2013. All chimeric tags were removed using the UCHIME algorithm and finally obtained effective tags for further analysis (Edgar et al., 2011). Sequences supporting this research which were published at the National Center for Biotechnology Information (NCBI) and the BioProject accession numbers were PRJNA762648 (bacterial 16S) and PRJNA762654 (fungal ITS1), respectively.

### Data Analysis of Microbial Diversity

The comparison of alpha diversity (α-diversity) among groups was analyzed using the Welch’s *t*-test and Wilcoxon rank test in the R project Vegan package (version 2.5.3). The comparison of α-index among groups was calculated using the Kruskal–Wallis H test and Tukey’s HSD test in the R project Vegan package (version 2.5.3). Thereinto, Shannon, Chao1, and ACE were computed in QIIME (version 1.9.1) (Caporaso et al., 2010). The principal component analysis (PCA) was constructed by the R project Vegan package (version 2.5.3) to perform beta diversity (β-diversity), and the significance was determined by ANOSIM. Meanwhile, the abundance statistics of taxonomy was visualized using Krona (version 2.6) (Ondov et al., 2011). Heatmap and circular layout representations of species abundance were graphed using pheatmap package (version 1.0.12) in R project (Kolde, 2015) and circus (version 0.69-3) (Krzywinski et al., 2009), respectively.

### Inhibitory Activity of *Fusarium oxysporum* f. sp. *niveum* Bioassays

The FON strain was cultured on potato dextrose agar (PDA) media (BD Difco/BBL, BD-213200). To study the mycelium inhibitory effects, different concentrations of SAEW were added to the same initial concentration of FON suspension liquid and reacted for 60 s. Later, the mixed liquor was cultivated in 28°C and 180 r/min shakers for 5 days. The FON was collected by 10,000 r/min centrifugation for 10 min to measure the wet weight, and the dry weight of FON was detected after mycelium dried in a 60°C baking oven for 48 h. In addition, the suspension liquid of FON was mixed with an equal volume of concentrated sulfuric acid and 0.5% anthranone. After having a boiling water bath for 10 min, the mixed liquid was cool down to room temperature and detected absorbance *via* a spectrophotometer. Finally, the sugar content of FON mycelium was calculated using a formula as follows: the sugar content = (*A* × *V*)/(Vt × *M* × 10^6^) × 100%. *A* and *V* denote the sugar content on the standard curve and the total volume of liquid, respectively. Vt and *M* were the volume and weight of the sample.

Notably, 10 g dry continuous cropping soil was added into 90 ml sterile water, which was cultivated in 180 r/min shakers for 60 min. The supernatant was collected and diluted with different concentrations of SAEW. Finally, 200 μl of mixed liquor was taken and coated on the FON cultivated medium. The number of colonies was counted when the medium plate was cultivated in a 28°C thermostatic incubator for 3 days. The number of colony (CFU/g) = the mean number of colony in diluent × dilution ratio/dry weight. Bacteriostasis rate = (the number of colony in Con-the number of colony in treated group)/the number of colonies in Con × 100%. The correlation between watermelon growth index, FON, and SAEW application was analyzed using R language (R 3.5.2, the latest version of the R language for statistical computation and graphics).

### The Analyses of Reactive Oxygen Species Content in *Fusarium oxysporum* f. sp. *niveum*

The ROS level of FON was detected using fluorescence probe DCFH-DA (2,7-dichlorodi-hydrofuorescein diacetate, Sigma-Aldrich, CAS:4091-99-0). The levels of ROS were measured by fluorescence intensity. After being treated with different concentrations of SAEW for 5 min, the samples were centrifuged at 8,000 rpm for 10–15 min in a refrigerated centrifuge (4°C). Then, the samples were resuspended in 1,000 μl phosphate-buffered saline (PBS) and incubated at 37°C in the dark for 30 min blending with 10 μM DCFH-DA working solution. PBS was used as blank control. Approximately 10,000 cells were gathered for each sample to detect the ROS contents by flow cytometry (FCM, BD Accuri C6 Plus, United States).

### Statistical Analysis

Statistical analyses were constructed using SPSS statistical software (version 11.0). The significant differences between treatments were analyzed through the one-way analysis of variance (ANOVA) and Duncan’s multiple range test. *P* < 0.05 was used as the threshold. The normality test of all data was verified before significance analysis (*P* > 0.05). All the data in this study were measured with three independent experiments.

## Results

### Growth Situation of Watermelon Seedlings Influenced by Slightly Acidic Electrolyzed Water

To figure out the effect of SAEW on the growth of plants, we treated watermelon seedlings with different concentrations of SAEW. As shown in Supplementary Figure 2, all growth indices were developed with varying degrees as the SAEW concentration increased. Thereinto, SAEW60 could significantly promote the growth of watermelon seedlings grown in continuous cropping soil. Compared with Con, the shoot fresh weight, root fresh weight, and shoot dry weight of SAEW60-treatment were increased by 15.62%, 14.72%, and 24.46%, respectively. However, biomass improvement had no significant differences between SAEW60 and SAEW80. Photosynthetic efficiency is a crucial indicator to assess the situation of plant growth. Our analyses of photosynthetic parameters revealed that the photosynthetic efficiency was gradually raised with the concentration of SAEW increase (Supplementary Figure 3). Compared with the Con, SAEW60 could severally assess the concentration of intercellular CO_2_, net photosynthetic rate, stomatal conductance, and transpiration rate by 18.42%, 52.08%, 33.33%, and 33.34%, respectively. SAEW80 further increased these photosynthetic parameters but with no significance to SAEW60 treatment. In addition, as the SAEW-treated concentrations increased, the chlorophyll fluorescence parameters were gradually improved (Supplementary Figure 4). The PSII photochemical efficiency, photochemical quenching coefficient, non-photochemical quenching coefficient, and photosynthetic electron transport rate were separately increased by 35.29%, 36.84%, 27.5%, and 31.25% with SAEW60 treatment, which was in accordance with the change tendency of photosynthetic parameters. In brief, SAEW could greatly improve the growth situation of watermelon seedlings, and 60 ppm SAEW was selected as the optimum to proceed the further research.

### Slightly Acidic Electrolyzed Water Had the Capacity for Changing the Fungal and Bacterial Communities in Watermelon Continuous Cropping Soil

**FIGURE 1:**
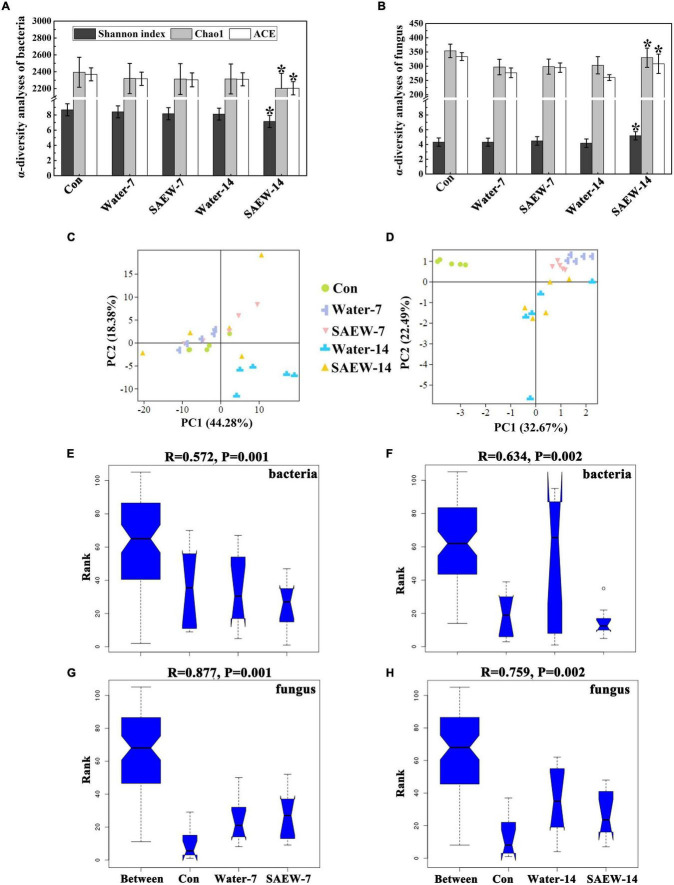
The α- and β-diversity analyses of bacteria and fungus in each treatment. **(A,B)** show the α-diversity analyses of bacteria and fungus, respectively. **(C,D)** represent the β-diversity analyses (principal component analysis, PCA) of fungus and bacteria. The PCA plot is based on Bray–Curtis distance at the operational taxonomic unit (OTU) level. Significance was determined by ANOSIM and is shown in panels **(E–H)**. **(E,F)** show the bacteria of different treatments in 7 and 14 days. **(G,H)** show the fungus of different treatments in 7 and 14 days. The soils with different treatments (Con, Water-7, Water-14, SAEW-7, and SAEW-14) were separately collected from 5 replicated pots for each. Con and control (dry soil); Water-7 (the soil irrigated with deionized water for 7 days); Water-14 (the soil irrigated with deionized water for 14 days); SAEW-7 (the soil irrigated with 60 ppm concentration of slightly acidic electrolyzed water for 7 days); SAEW-14 (the soil irrigated with 60 ppm concentration of slightly acidic electrolyzed water for 14 days).

The fungal α-diversity index (Shannon, Chao 1, and ACE index) was both decreased by Water- and SAEW-treatments compared with the Con (original continuous cropping soil), which may arise from the microbial loss induced by irrigation of water and SAEW. In bacteria, the Shannon, Chao 1, and ACE index of SAEW-7 and SAEW-14 were lower than that of the Water-treatments (Figure 1A). However, the α-diversity of fungus exhibited an opposite relationship. The fungal α-diversity of SAEW-treatment was higher than that of the Water-treatment (Figure 1B), whether treated for 7 or 14 days. Then, the β-diversity analysis (PCA) was implemented for bacteria and fungus (Figures 1C,D). In terms of bacteria, the two main coordinates extracted explained 62.66% of the variation, of which 44.28% were explained by PC1 and 18.38% by PC2 (Figure 1C). Besides, the distance between Con, Water-7, and SAEW-7 was small and they gathered together. But SAEW-14 was far away from Water-14. When it came to fungus (Figure 1D), the two main coordinates extracted explained 55.16% of the variation, of which 32.67% were explained by PC1 and 22.49% by PC2. It showed that the samples of Con were far away from those collected in the Water- and SAEW-treatments, and the distance between Water-7 and SAEW-7 was small. In addition, the five samples of Con, Water-7, and SAEW-7 were gathered together and not dispersed as the samples of Water-14 and SAEW-14. The significance analysis of microbial community composition (PCA) differences was further determined by ANOSIM. The boxplot revealed that the microbial diversity of each treated group was significantly different from another one (ANOSIM: bacteria in 7 days, *R* = 0.572, *P* = 0.001; bacteria in 14 days: *R* = 0.634, *P* = 0.002; fungus in 7 days, *R* = 0.877, *P* = 0.001; fungus in 14 days, *R* = 0.759, *P* = 0.002) (Figures 1E-H), which indicated that SAEW could remarkably change the microbial diversity of continuous cropping soil, especially for fungus. All the results suggested that SAEW ameliorated the unbalanced soil microbial community structure by increasing fungal diversity while reducing bacterial diversity.

**FIGURE 2:**
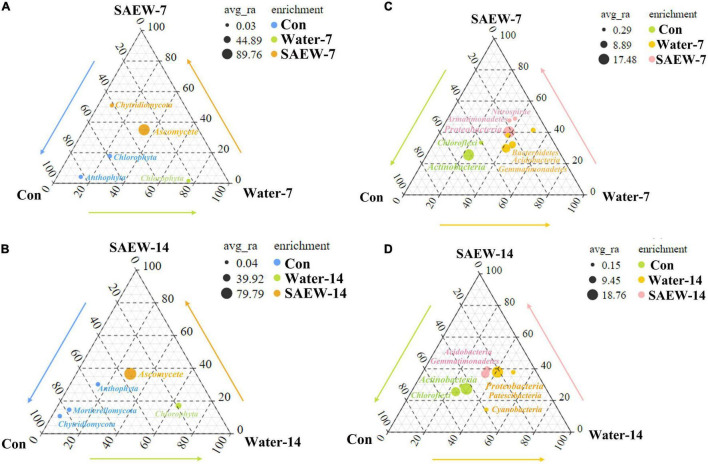
Ternary plot analysis of the indicator species of different treatments of fungus **(A,B)** and bacteria **(C,D)**. The top key species (OTUs) in each treatment were labeled in the corresponding color. The soils with different treatments (Con, Water-7, Water-14, SAEW-7, and SAEW-14) were separately collected from 5 replicated pots for each. Con and control (dry soil); Water-7 (the soil irrigated with deionized water for 7 days); Water-14 (the soil irrigated with deionized water for 14 days); SAEW-7 (the soil irrigated with 60 ppm concentration of slightly acidic electrolyzed water for 7 days); SAEW-14 (the soil irrigated with 60 ppm concentration of slightly acidic electrolyzed water for 14 days).

To figure out the indicator fungal and bacterial species affected by SAEW, the ternary plot (Figure 2 and Supplementary Tables 1-4) of species distribution was constructed. As shown in Figure 2, the ternary plot analysis was carried out for bacteria and fungus according to the OTUs of every species. For fungus (Figure 2A), *Ascomycete* and *Chytridiomycota* were considered as the dominant phyla in SAEW-7. Meanwhile, the Water-7 treatment contained only one dominant phylum called *Chlorophyta*. *Anthophyta* and *Chlorophyta* were the dominant phyla in Con. The enrichment of each species and their ratio in each treatment were described in detail in Supplementary Tables 1, 2. When the treated time was extended to 14 days, the corresponding dominant phylum in each treatment was changed slightly in Con and SAEW-14 (Figure 2B). Specifically, *Anthophyta*, *Mortierellomycota*, and *Chytridiomycota* were dominantly clustered in Con, while *Ascomycete* became the only dominant phylum in SAEW-14. *Chlorophyta* was still the dominant phylum in Water-14. When it came to bacteria, the dominant phyla in each treatment were changed between 7 and 14 days (Figures 2C,D). The dominant phyla of Water-7 were *Bacteroidetes*, *Acidobacteria*, and *Gemmatimonadetes*, and the dominant phyla of Water-14 became *Proteobacteria*, *Patescibacteria*, and *Cyanobacteria*. In addition, *Nitrospirae*, *Armatimonadetes*, and *Proteobacteria* were the dominant phyla in SAEW-7, and *Acidobacteria* and *Gemmatimonadetes* were the dominant phyla in SAEW-14. *Actinobacteria* and *Chloroflexi* were the dominant phyla in Con. The enrichment of each bacterial species and their ratio in each treatment are described in detail and shown in Supplementary Tables 3, 4.

**FIGURE 3:**
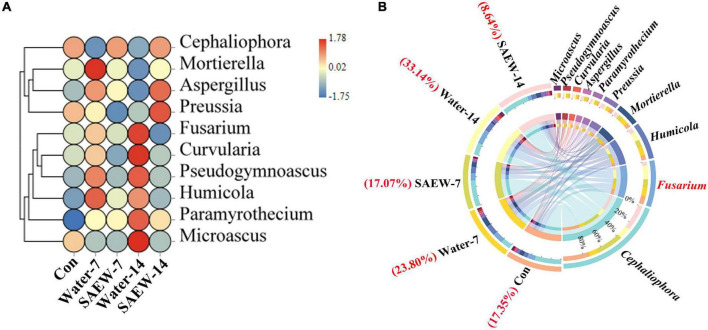
Heatmap and Circos analyses of the key fungal species that were significantly affected by slightly acidic electrolyzed water (SAEW). **(A)** shows the heatmap of species distribution at the genus level according to OTUs, and **(B)** shows the ratios of the main fungal species to the fungal amount. The more red, the higher abundance of species, and the more blue, the lower abundance of species in panel **(A)**. The red font of panel **(B)** represents the ratio of *Fusarium* of different treatments. The soils with different treatments (Con, Water-7, Water-14, SAEW-7, and SAEW-14) were separately collected from 5 replicated pots for each. Con and control (dry soil); Water-7 (the soil irrigated with deionized water for 7 days); Water-14 (the soil irrigated with deionized water for 14 days); SAEW-7 (the soil irrigated with 60 ppm concentration of slightly acidic electrolyzed water for 7 days); SAEW-14 (the soil irrigated with 60 ppm concentration of slightly acidic electrolyzed water for 14 days).

The further diversity analyses of fungus at the genus level according to OTUs revealed that *Cephaliophora*, *Fusarium*, *Humicola*, *Mortierella*, *Preussia*, *Paramyrothecium*, *Aspergillus*, *Curvularia*, *Pseudogymnoascus*, and *Microascus* were the top 10 clustered fungal genera by heatmap and Circos analyses (Figure 3). The abundances of *Fusarium*, *Curvularis*, *Pseudogymnoascus*, and *Humicola* involved in biotic stress were significantly decreased by SAEW-7 and SAEW-14 compared with Water-7 and Water-14, respectively, in the heatmap (Figure 3A). Thereinto, the change fold of *Fusarium* between Water- and SAEW-treatments was more than other fungal species. Meanwhile, the detailed ratio of *Fusarium* with different treatments to the fungal amount was analyzed by Circos (Figure 3B). It revealed that the ratio of *Fusarium* was reduced by SAEW, which was lowered from 23.8% in Water-7 to only 17.07% in SAEW-7. Similarly, the ratio of *Fusarium* was reduced from 33.14% in Water-14 to 8.64% in SAEW-14. It indicated that SAEW could obviously decrease the ratio of *Fusarium* in the microbial community, which may finally reduce the *Fusarium* wilt disease incidence of watermelon induced by continuous cropping. In terms of bacteria, the heatmap analysis revealed that 20 species were the main clustered species. Thereinto, *Iamia*, *Streptomyces*, *Allorhizobium*, and *Brevundimonas* were remarkably reduced but *Pirellula*, *Pir4-lineage*, and *Stenotrophobacter* were increased by SAEW (Supplementary Figure 5A). The further Circos analysis (Supplementary Figure 5B) performed that *Gemmatimonas*, *Sphingomonas*, *Blastococcus*, *Ellin6055*, *Pir4-lineage*, *Nocardioides*, *Microvirga*, *Haliangium*, and *Marmoricola* occupied a large proportion in total bacteria. However, compared with the species distribution of fungus, the bacteria involved in biotic stress had no significant differences between Water- and SAEW-treatments. That is, the effect of SAEW on fungus was greater than it on bacteria.

### The Relative Abundance and Intracellular Reactive Oxygen Species Concentration of *Fusarium* Were Significantly Influenced by Slightly Acidic Electrolyzed Water

**FIGURE 4:**
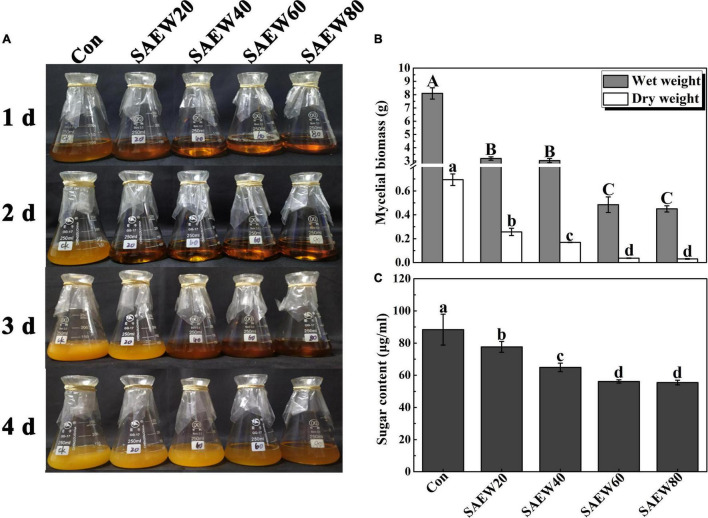
Effects of SAEW on the growth of *Fusarium oxysporum* f. sp. *niveum* (FON) mycelium. **(A)** shows the phenotype of the FON mycelium with different treatments. **(B,C)** show the mycelium biomass and sugar content of FON mycelium, respectively. Different concentrations of SAEW were added to the same initial concentration of FON suspension liquid. Vertical bars represent the standard deviation (SD) of the mean of nine biological replicates (*n* = 9). The data were collected from three independent experiments with three biological replicates for each. Different letters indicate significant differences at *P* < 0.05 according to the Duncan’s multiple range test. Con and control (deionized water); SAEW20 (20 ppm concentration of slightly acidic electrolyzed water); SAEW40 (40 ppm concentration of slightly acidic electrolyzed water); SAEW60 (60 ppm concentration of slightly acidic electrolyzed water); SAEW80 (80 ppm concentration of slightly acidic electrolyzed water); FON, *Fusarium oxysporum* f. sp. *Niveum*.

To verify the fungistasis of SAEW to FON, the mycelial biomass and the sugar content of FON cultivated in a fluid medium were first explored (Figure 4). The phenotypic analysis showed that the fluid medium became more and more limpid as the concentration of SAEW increased, and the obvious sterilization could last for 4 days of 60 and 80 ppm SAEW (Figure 4A). According to the analyses of mycelial fresh and dry weight, SAEW60 and SAEW80 could further inhibit the growth of FON mycelium. Compared with the Con, the mycelial wet weight was decreased by 93.83% and 94.20% with SAEW60 and SAEW80 treatments, respectively. Similarly, the mycelial dry weight of SAEW60 and SAEW80 was separately lowered by 92.86% and 93.57% compared with the Con (Figure 4B). Moreover, the sugar contents were gradually reduced by SAEW (Figure 4C). SAEW60 and SAEW80 could dramatically decrease the sugar content of FON mycelium by 32.58% and 33.71%, which revealed that the death of FON was prompted by SAEW.

**FIGURE 5:**
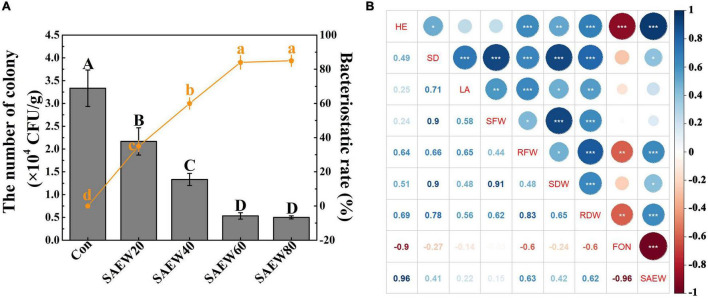
Effects of SAEW on the growth of FON in continuous cropping soil and the correlation analyses of watermelon growth indices, FON, and SAEW application. **(A)** shows the number of the FON colony and corresponding bacteriostasis rate of different treatments. The correlation between watermelon growth indices, FON, and SAEW application is shown in **(B)**. The number of the colony (CFU/g) = the mean number of the colony in diluent × dilution ratio/dry weight. Bacteriostasis rate = (the number of the colony in Con-the number of the colony in treated group)/the number of the colony in Con × 100%. Data are means ± SE from three independent experiments. Vertical bars represent the standard deviation (SD) of the mean of 9 biological replicates (*n* = 9). Additionally, bars with different lowercase letters are the significant differences between the bacteriostasis rate of Con and it of treated groups at *P* < 0.05 according to the Duncan’s multiple range test. The correlation analyses were conducted using R language with the watermelon growth indices in Supplementary Figure 2 and the number of FON colonies in soil with treatments of different SAEW concentrations in **(A)**. The asterisk in **(B)** reveals the significant differences in correlation. Blue and red indicate the positive and negative correlations, respectively. Data are means ± SE from three independent experiments with 5 biological replicates for each (*n* = 15). *, **, and *** represent significant differences at *P* < 0.05, 0.01, and 0.001, respectively. Con and control (deionized water); SAEW20 (20 ppm concentration of slightly acidic electrolyzed water); SAEW40 (40 ppm concentration of slightly acidic electrolyzed water); SAEW60 (60 ppm concentration of slightly acidic electrolyzed water); SAEW80 (80 ppm concentration of slightly acidic electrolyzed water); FON, *Fusarium oxysporum* f. sp. *niveum*; HE, height; SD, stem diameter; LA, leaf area; SFW, shoot fresh weight; RFW, root fresh weight; SDW, shoot dry weight; RDW, root dry weight.

Subsequently, the suppression effect of SAEW on the growth of FON in continuous cropping soil was further explored (Figure 5A). As the concentration of SAEW increased, the number of colonies was reduced by degrees. Compared with the Con, the number of colonies was decreased by 84.85% with SAEW60 treatment, while the bacteriostatic rate was reached almost 83.56% with SAEW60 treatment. Similarly, SAEW80 had a great capacity for the inhibition of FON, which had no significant difference with SAEW60. In conclusion, the growth of FON could be dramatically inhibited by SAEW. All the conclusions suggested that SAEW assuredly had the capacity for fungistasis to FON, which was finally beneficial to improving the growth of watermelon seedlings. Moreover, the correlation between watermelon growth index, FON, and SAEW application was analyzed using the R language (Figure 5B). The watermelon growth indices (Supplementary Figure 2) and the number of FON colonies in soil with treatments of different SAEW concentrations (Figure 5A) were used to conduct the correlation analysis. It revealed that all the growth indices had a negative correlation with FON, especially for height (HE, -0.9), shoot fresh (SFW, -0.6), and dry (SDW, -0.6) weights (Figure 5B). In addition, there was a strong negative correlation between SAEW and FON (-0.96). On the contrary, the watermelon growth indices had a strong positive correlation with SAEW application including HE with 0.96, SFW with 0.63, and SDW with 0.62. All the conclusions suggested that there was a strong correlation between watermelon, FON, and SAEW.

**FIGURE 6:**
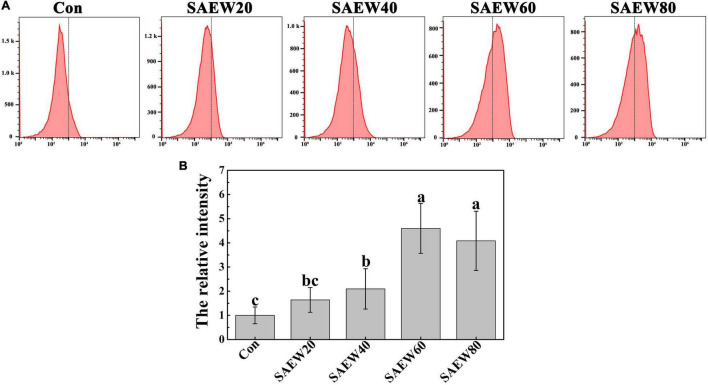
The effect of SAEW on the ROS production in FON. **(A)** The DCF fluorescence of different treatments was measured by flow cytometry. **(B)** The relative intensity of DCF fluorescence was calculated by the ratio of different concentrations of SAEW to Con. Data are means ± SE from three independent experiments with 3 biological replicates for each (*n* = 9). Different letters indicate significant differences at *P* < 0.05 according to the Duncan’s multiple range test. Con and control (deionized water); SAEW20 (20 ppm concentration of slightly acidic electrolyzed water); SAEW40 (40 ppm concentration of slightly acidic electrolyzed water); SAEW60 (60 ppm concentration of slightly acidic electrolyzed water); SAEW80 (80 ppm concentration of slightly acidic electrolyzed water); ROS: Reactive oxygen species; FON: *Fusarium oxysporum* f. sp. *niveum*.

To further verify the sterilizing effect of SAEW on FON, the relative fluorescence intensity was assessed by the ratio of fluorescence value in SAEW-treated groups to it in Con. As shown in Figure 6A, the intracellular DCF fluorescence was gradually improved with the concentration of SAEW increasing. Thereinto, the fluorescence intensity was increased over 4-fold at SAEW60 treatment compared with the Con. All the observations indicated that SAEW could induce the intracellular ROS to redundantly accumulate, which finally made the FON suffered from a heavy oxidative stress.

## Discussion

**FIGURE 7:**
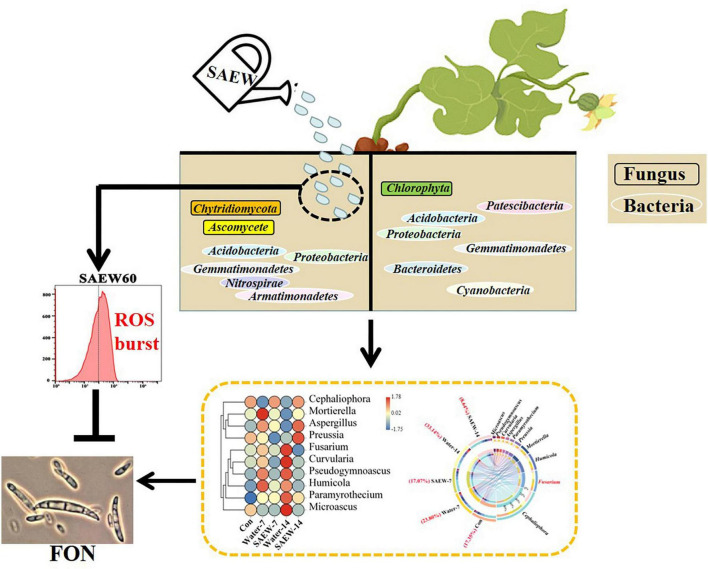
The mechanism model of SAEW alleviated watermelon continuous cropping obstacle. The left and the right halves of soil in the graph show the soil with or without SAEW treatment, respectively. SAEW could significantly improve the growth of watermelon seedlings grown in continuous cropping soil. The bacterial diversity in soil was reduced but the fungal diversity was induced by SAEW. The corresponding indicator species are marked in the model. The white oval and black rectangle separately represent the bacterial and fungal indicator species of different treatments. Thereinto, the proportion of FON was remarkably decreased by SAEW. The sterilization effect of SAEW was further verified by the analyses of FON mycelial growth, which may arise from the intracellular ROS production was greatly induced by SAEW treatment. ROS: Reactive oxygen species; FON: *Fusarium oxysporum* f. sp. *niveum*.

Crop failure and economic losses of watermelon have resulted from continuous cropping obstacle (Wang et al., 2019; Huang et al., 2021). Continuous cropping obstacle is generated by a mass of biotic and abiotic factors including the deteriorated properties of soil physicochemical characters (Zydlik and Zydlik, 2013; Kaur and Singh, 2014; Chen et al., 2020; Li et al., 2021), autotoxicity of plants (van Wyk et al., 2017; Cheng et al., 2020), and changes of the microbial diversities (Dong et al., 2017; Sui et al., 2019; Gao et al., 2021; Jie et al., 2021; Xia et al., 2021). Our results confirmed that applying SAEW could alleviate the growth inhibition of watermelon seedlings induced by continuous cropping obstacle (Figure 7). It showed that SAEW influenced the microbial community in soil and inhibited the growth of FON by stimulating intracellular ROS accumulation, which may contribute to the growth of watermelon. The application of SAEW provided an efficient and convenient technique to relieve watermelon continuous cropping obstacle, which could increase ecological sustainability in the industrial chain of facility watermelon production. SAEW has been used in fruit and vegetable industry arising from its high bactericidal efficacy with a minimal damage to crop produce, equipment, and environment (Hao J. et al., 2011; Ding et al., 2015; Xuan et al., 2016). Besides, our previous research has confirmed that SAEW not only disinfected watermelon seeds but also significantly improved the germination of triploid watermelon seeds which delayed seed germination and irregular emergence frequently occurred (Wu et al., 2022).

### Slightly Acidic Electrolyzed Water Could Significantly Improve the Growth Watermelon Seedlings Which Were Cultivated in Continuous Cropping Soil

As the SAEW concentration increased, the growth situation of watermelon seedling was improved (Supplementary Figure 2). Compared with Con, SAEW60 had a capacity for promoting the growth of watermelon seedlings, especially for shoot fresh weight, root fresh weight, and shoot dry weight (Supplementary Figure 2). Some studies have revealed that continuous cropping obstacle gave rise to reducing photosynthetic efficiency in watermelon (Everts et al., 2014; Li H. et al., 2019). In this research, the photosynthetic parameters (Supplementary Figure 3) and chlorophyll fluorescence parameters (Supplementary Figure 4) were greatly improved by SAEW60-treatment. In short, SAEW60 could alleviate the weakened photosynthetic efficiency of watermelon induced by continuous cropping obstacles.

### The Changes of Microbial Diversity Were Involved in the Alleviation of Continuous Cropping Obstacle in Watermelon Seedlings Induced by Slightly Acidic Electrolyzed Water

Microbial diversity is a crucial property that can not only reflect the fertility and health of soil but also be beneficial to estimate the growth of crops (Congreves et al., 2015; Lemaire et al., 2019; Ding et al., 2021). The interpretations of continuous cropping obstacle are diverse but the deterioration of microbial diversity is one of the main reasons (Zhang et al., 2020; Chen et al., 2022). Existing studies have shown that microorganisms of continuous cropping soil were altered by intercropping crops (Fang et al., 2018; Li et al., 2018, 2020; Zeng et al., 2020; Tang et al., 2021), application of urban waste compost, corn straw, biochar, and vermicompost (Gao et al., 2020; Ding et al., 2021; Wang et al., 2021). SAEW was proven to be effective on sterilization in fruit and vegetable industry (Hao et al., 2021). However, the effect of SAEW on the microbial communities has not been reported so far. In this study, the fungal and bacterial α-diversities in Water- and SAEW-treatments were changed by degrees compared with Con (Figures 1A,B). β-diversities also indicated that SAEW had a strong capacity for altering the fungal and bacterial community structure (Figures 1C,H). Some previous studies have shown that long-term continuous monoculture reduced fungal diversity while raised bacterial diversity, which led to the unbalanced soil microbial community structure in watermelon, silva, and potato (Rosenzweig et al., 2012; Tang et al., 2014; Wang et al., 2019). In other words, SAEW could recover the disequilibrium of soil microbial community induced by the continuous monoculture of watermelon by enhancing the fungal α-diversity but decreasing the bacterial α-diversity. The analyses of microbial diversity revealed that indicator fungal and bacterial species were changed with different treatments (Figure 2). Thereinto, *Ascomycete* and *Chytridiomycota* were the dominant fungal phyla in SAEW-treatments. *Chlorophyta* was the only dominant fungal phylum in Water-treatments. Besides, the change of indicator bacterial species was large than fungus. *Bacteroidetes*, *Acidobacteria*, *Gemmatimonadetes*, *Proteobacteria*, *Patescibacteria*, and *Cyanobacteria* were the dominant bacterial phyla of Water-treatments. Meanwhile, *Nitrospirae*, *Armatimonadetes*, *Proteobacteria*, *Acidobacteria*, and *Gemmatimonadetes* were the dominant bacterial phyla in SAEW-treatments. The further heatmap and Circos analyses shown that *Fusarium* was the key fungus which was remarkably suppressed by SAEW (Figure 3). It suggested that SAEW could obviously decrease the ratio of *Fusarium* in microbial community, which may finally reduce the *Fusarium* wilt disease incidence of watermelon induced by continuous cropping. However, the change fold among bacterial species with different treatments was less than it in fungi (Supplementary Figure 5A). That is, the effect of SAEW on fungus was greater than it on bacteria.

### *Fusarium oxysporum* f. sp. *niveum* Was Observably Sterilized by Slightly Acidic Electrolyzed Water

The infection of FON, which was known as the most destructive soil-borne disease in watermelon, could give rise to severe limitation of watermelon production (Li et al., 2020; Ding et al., 2021). Proteomic analysis had revealed that FON led to the significant changes in expression of abundant proteins, and the potential candidate proteins involved in stress or defense had great relevance to the biological pathways which linked to the watermelon-FON pathosystem (Zhang et al., 2018). Besides, FON was identified as the most prevalent fungal strain which was isolated from the root of grafted watermelon infected by root rot and generated an increasing death rate of grafted watermelon seedlings (Zhang et al., 2021a,b). The sugar concentrations in root exudates of varied watermelon varieties could induce the diverse growth situation of watermelon by changing the growth of FON in rhizosphere (Hao W. et al., 2011; Ma et al., 2021). Therefore, FON and the effective measures to inhibit the growth of FON were the key research objects in watermelon production. To further verify the fungicidal effect of SAEW to FON, the changes of FON growth were investigated (Figure 4). It revealed that the growth of mycelium was gradually inhibited as the concentration of SAEW increased (Figure 4A). In addition, the mycelial weight and sugar content were significantly reduced by SAEW (Figures 4B,C). Similarly, the FON in continuous cropping soil was obviously decreased by SAEW, which the number of FON colony was inhibited by almost 83.56% with SAEW60 treatment (Figure 5A). The correlation analyses further indicated that there was a great positive correlation between watermelon growth indices and SAEW. However, a strong negative correlation was performed between SAEW and FON, which was in accordance with the correlation between FON and watermelon growth indices. Likewise, application of dolomitic limestone and wheat root exudates generated by wheat/watermelon companion cropping system could alleviate the watermelon continuous cropping obstacles *via* inhibiting the growth of FON (Xu et al., 2015; Lv et al., 2018; Li et al., 2020; McDonald et al., 2021). Taken together, the results suggested that SAEW assuredly had the capacity for fungistasis to FON, which was finally beneficial to improving the growth of watermelon seedlings. On account of the available chlorine of SAEW, we speculated that FON mycelium was suffered from oxidative stress. Moreover, a previous study had revealed that the growth of *E. coli* was inhibited by the abundant ROS accumulation induced by SAEW (Liao et al., 2017; Ye et al., 2017). The DCF fluorescence intensity was proportional to the amount of intracellular ROS (Liu et al., 2013; Ye et al., 2017). Our analyses of flow cytometry revealed that the intracellular DCF fluorescence was notably induced by SAEW (Figure 6), which was consistent with the effect of SAEW on *E. coli* (Ye et al., 2017). Therefore, the inhibited-growth of FON may arise from the ROS accumulation caused by SAEW.

## Conclusion

This study revealed the effects of SAEW on the growth of watermelon, soil microbial diversity, the sterilization to FON mycelium, and the ROS production in FON cells. It indicated that SAEW alleviated the watermelon continuous cropping obstacle by changing the diversity of soil microbial community and especially for depressing the growth of FON. In addition, the intracellular ROS production of FON was signally induced by SAEW, which led to the FON suffering from oxidative stress. SAEW is beneficial for the development of an economical and environment-friendly watermelon industry from seed germination to seedling cultivation. Therefore, SAEW has the potential to be a convenient, efficient, and environmentally friendly measure to solve the crop failure of watermelon. Future studies should be conducted on the application of SAEW to more crop existed continuous cropping obstacles, which will be helpful for the sustainable development of agriculture.

## Data Availability Statement

The datasets presented in this study can be found in online repositories. The names of the repository/repositories and accession number(s) can be found in the article/Supplementary Material.

## Author Contributions

XW and KC contributed to the conception of the study. XW designed and performed the experiments, analyzed the data, and wrote the manuscript. CW, DL, and YW helped with graphical edit. ZY, LX, and EB critically reviewed the manuscript. All authors contributed to the article and approved the submitted version.

## Conflict of Interest

The authors declare that the research was conducted in the absence of any commercial or financial relationships that could be construed as a potential conflict of interest.

## Publisher’s Note

All claims expressed in this article are solely those of the authors and do not necessarily represent those of their affiliated organizations, or those of the publisher, the editors and the reviewers. Any product that may be evaluated in this article, or claim that may be made by its manufacturer, is not guaranteed or endorsed by the publisher.

## Supplementary Material

Supplementary Figure 1 The photograph of the soil sampling point. **(A,B)** represent the different viewing perspectives of the soil sampling point.

Supplementary Figure 2 The effect of slightly acidic electrolyzed water (SAEW) on the growth index of watermelon seedlings cultivated in continuous cropping soil. Watermelon seedlings were treated with 0, 20, 40, 60, and 80 ppm SAEW. SAEW was applied every 3 days, and the growth indices were measured after the plants grew to four true leaves and a terminal bud. **(A,B)** show the fresh and dry weights of watermelon seedlings of different treatments, respectively; **(C)** represents the height and stem diameter. The leaf areas with different treatments were performed in **(D)**. Data are means ± SE from three independent experiments. Vertical bars represent the standard deviation (SD) of the mean of 45 biological replicates (*n* = 45). Bars with different letters are significantly different at *P* < 0.05 according to the Duncan’s multiple range test. SAEW, slightly acidic electrolyzed water.

Supplementary Figure 3 Effects of SAEW on the photosynthetic parameters of watermelon seedlings grown in continuous cropping soil. Watermelon seedlings were treated with 0, 20, 40, 60, and 80 ppm SAEW. SAEW was applied every 3 days, and the photosynthetic parameters were measured after the plants were grown to four true leaves and a terminal bud. **(A)** shows the intercellular CO_2_ concentration of different treatments; **(B–D)** represent the net photosynthetic rate, stomatal conductance, and transpiration rate, respectively. Data are means ± SE from three independent experiments. Vertical bars represent the standard deviation (SD) of the mean of 15 biological replicates (*n* = 15). Bars with different letters are significantly different at *P* < 0.05 according to the Duncan’s multiple range test. SAEW, slightly acidic electrolyzed water; CO_2_, carbon dioxide.

Supplementary Figure 4 Effects of SAEW on the chlorophyll fluorescence parameters of watermelon seedlings grown in continuous cropping soil. Watermelon seedlings were treated with 0, 20, 40, 60, and 80 ppm SAEW. **(A)** shows the PSII photochemical efficiency; **(B–D)** represent the photochemical quenching coefficient, non-photochemical quenching coefficient, and photosynthetic electron transport rate, respectively. Data are means ± SE from three independent experiments. Vertical bars represent the standard deviation (SD) of the mean of 15 biological replicates (*n* = 15). Bars with different letters are significantly different at *P* < 0.05 according to the Duncan’s multiple range test. SAEW, slightly acidic electrolyzed water; PSII, photosystem II.

Supplementary Figure 5 Heatmap and Circos analyses of the key bacterial species that were affected by SAEW. **(A)** shows the heatmap of species distribution at the genus level according to OTUs, and **(B)** shows the ratios of the main bacterial species to the total bacteria. The more red, the higher abundance of species, and the more blue, the lower abundance of species in panel **(A)**. The soils with different treatments (Con, Water-7, Water-14, SAEW-7, and SAEW-14) were separately collected from 5 replicated pots for each. Con and control (dry soil); Water-7 (the soil irrigated with deionized water for 7 days); Water-14 (the soil irrigated with deionized water for 14 days); SAEW-7 (the soil irrigated with 60 ppm concentration of slightly acidic electrolyzed water for 7 days); SAEW-14 (the soil irrigated with 60 ppm concentration of slightly acidic electrolyzed water for 14 days).

Supplementary Table 1 The ternary plot of indicator fungi in each treatment of 7 days. Note: the soils with different treatments (Con, Water-7, Water-14, SAEW-7, and SAEW-14) were separately collected from 5 replicated pots for each. Con and control (dry soil); Water-7 (the soil irrigated with deionized water for 7 days); SAEW-7 (the soil irrigated with 60 ppm concentration of slightly acidic electrolyzed water for 7 days).

Supplementary Table 2 The ternary plot of indicator fungi in each treatment of 14 days. Note: the soils with different treatments (Con, Water-7, Water-14, SAEW-7, and SAEW-14) were separately collected from 5 replicated pots for each. Con and control (dry soil); Water-14 (the soil irrigated with deionized water for 14 days); SAEW-14 (the soil irrigated with 60 ppm concentration of slightly acidic electrolyzed water for 14 days).

Supplementary Table 3 The ternary plot of indicator bacteria in each treatment 7 days. Note: the soils with different treatments (Con, Water-7, Water-14, SAEW-7, and SAEW-14) were separately collected from 5 replicated pots for each. Con and control (dry soil); Water-7 (the soil irrigated with deionized water for 7 days); SAEW-7 (the soil irrigated with 60 ppm concentration of slightly acidic electrolyzed water for 7 days).

Supplementary Table 4 The ternary plot of indicator bacteria in each treatment of 14 days. Note: the soils with different treatments (Con, Water-7, Water-14, SAEW-7, and SAEW-14) were separately collected from 5 replicated pots for each. Con and control (dry soil); Water-14 (the soil irrigated with deionized water for 14 days); SAEW-14 (the soil irrigated with 60 ppm concentration of slightly acidic electrolyzed water for 14 days).
