# Supplementary figures and images for: Variation of Soil Microbial Community and Sterilization to Fusarium oxysporum f. sp. niveum Play Roles in Slightly Acidic Electrolyzed Water-Alleviated Watermelon Continuous Cropping Obstacle
Source: Front Microbiol. 2022 Apr 28;13:837121. doi: 10.3389/fmicb.2022.837121 (PMC9097028; doi:10.3389/fmicb.2022.837121)

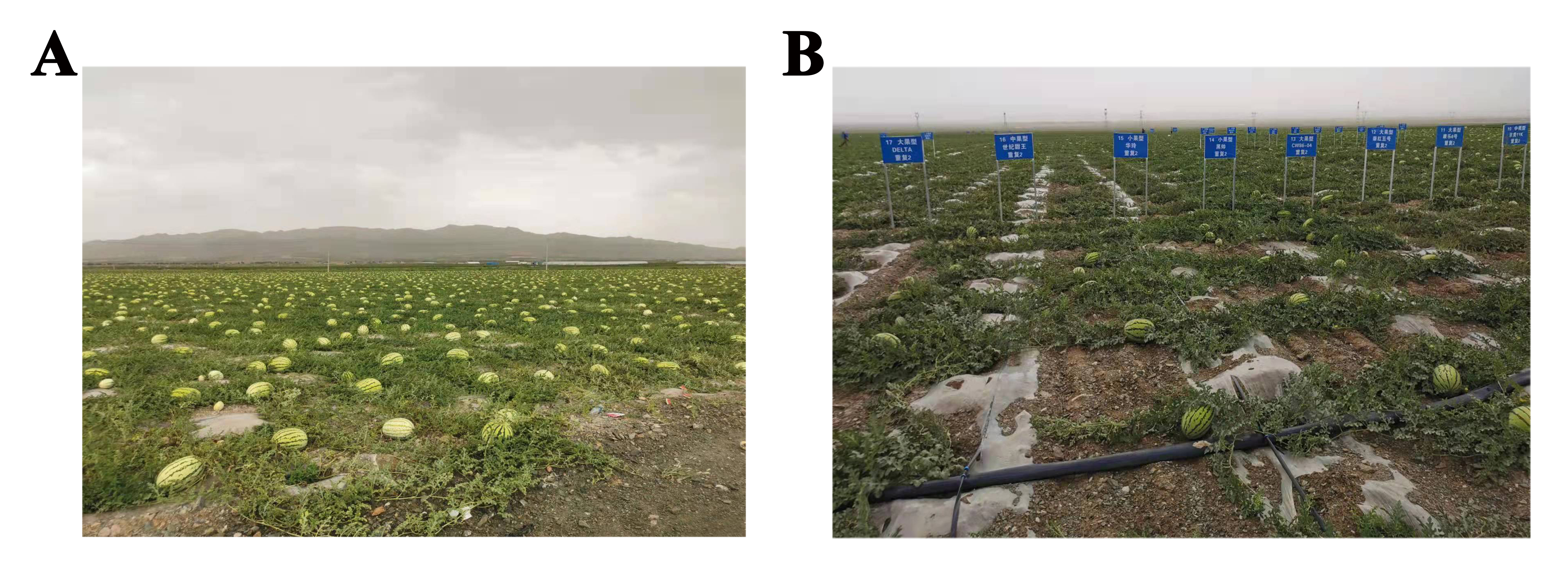

Supplement: Supplementary Figure 1 — The photograph of the soil sampling point. (A,B) represent the different viewing perspectives of the soil sampling point. [file Image_1.JPEG]

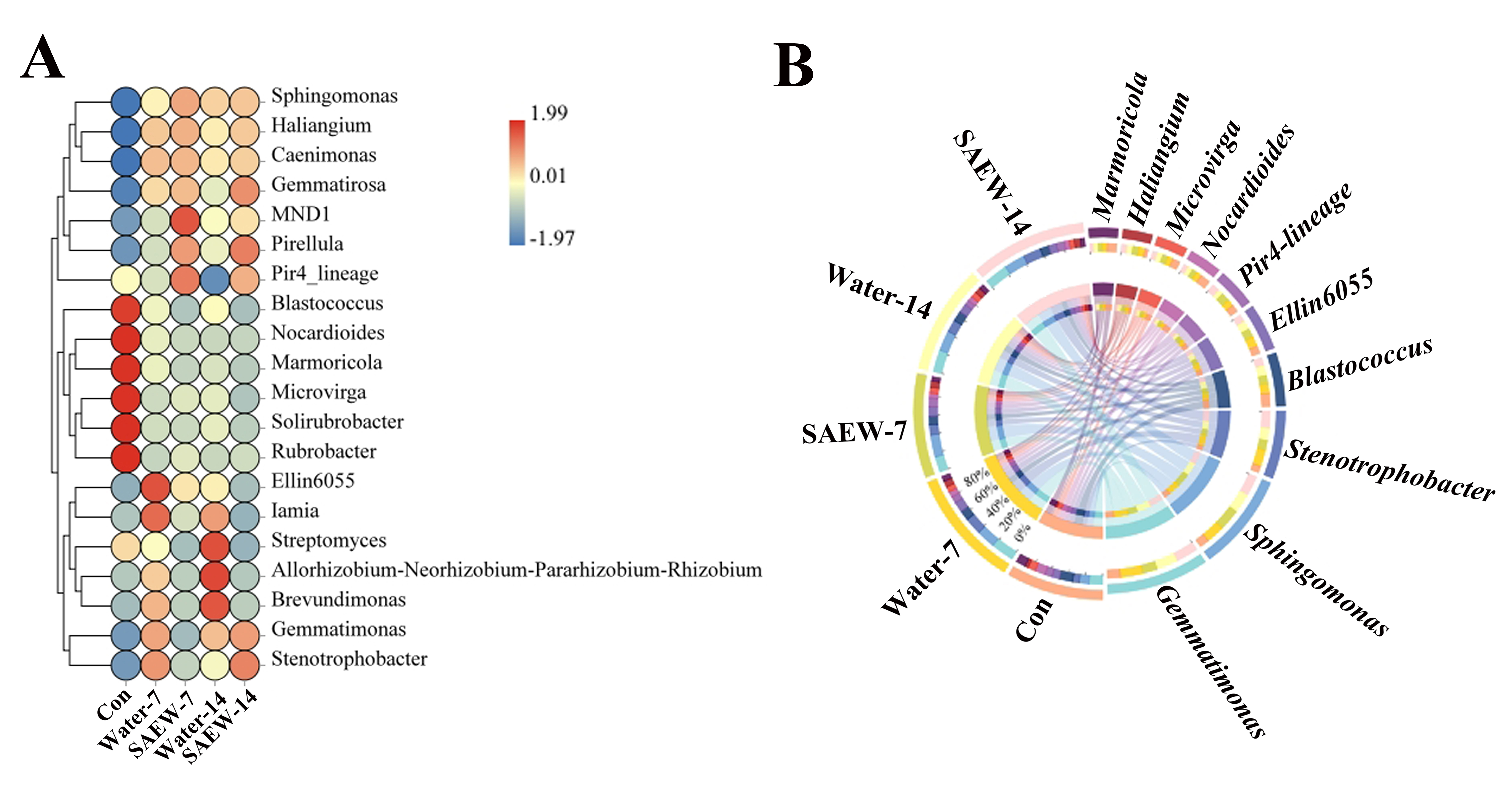

Supplement: Supplementary Figure 5 — Heatmap and Circos analyses of the key bacterial species that were affected by SAEW. (A) shows the heatmap of species distribution at the genus level according to OTUs, and (B) shows the ratios of the main bacterial species to the total bacteria. The more red, the higher abundance of species, and the more blue, the lower abundance of species in panel (A). The soils with different treatments (Con, Water-7, Water-14, SAEW-7, and SAEW-14) were separately collected from 5 replicated pots for each. Con and control (dry soil); Water-7 (the soil irrigated with deionized water for 7 days); Water-14 (the soil irrigated with deionized water for 14 days); SAEW-7 (the soil irrigated with 60 ppm concentration of slightly acidic electrolyzed water for 7 days); SAEW-14 (the soil irrigated with 60 ppm concentration of slightly acidic electrolyzed water for 14 days). [file Image_5.JPEG]
