# Supplementary material for: Variation of Soil Microbial Community and Sterilization to Fusarium oxysporum f. sp. niveum Play Roles in Slightly Acidic Electrolyzed Water-Alleviated Watermelon Continuous Cropping Obstacle
Source: Front Microbiol. 2022 Apr 28;13:837121. doi: 10.3389/fmicb.2022.837121 (PMC9097028; doi:10.3389/fmicb.2022.837121)
Supplement: Supplementary Table 1 — The ternary plot of indicator fungi in each treatment of 7 days. Note: the soils with different treatments (Con, Water-7, Water-14, SAEW-7, and SAEW-14) were separately collected from 5 replicated pots for each. Con and control (dry soil); Water-7 (the soil irrigated with deionized water for 7 days); SAEW-7 (the soil irrigated with 60 ppm concentration of slightly acidic electrolyzed water for 7 days). [file Table_1.DOC]

**Supplementary Table 1 The ternary plot of indicator fungi in each treatment of 7 d**

| Groups | Enrich | Enrichment | The ratio of  Con (%) | The ratio of  Water-7 (%) | The ratio of  SAEW-7 (%) | p-value | q-value |
| --- | --- | --- | --- | --- | --- | --- | --- |
| Anthophyta | Con | 5.2316 | 82.3003 | 13.5129 | 4.1868 | 0.0081 | 0.0163 |
| Chlorophyta | Con | 8.7835 | 59.0264 | 23.1729 | 17.8007 | 0.0045 | 0.0119 |
| Mucoromycota | Water-7 | 0.0173 | 22.4437 | 75.9772 | 1.5791 | 0.0040 | 0.0119 |
| Ascomycota | SAEW-7 | 94.3130 | 30.9213 | 34.0536 | 35.0250 | 0.0045 | 0.0119 |
| Chytridiomycota | SAEW-7 | 0.1214 | 41.2530 | 7.7853 | 50.9616 | 0.0263 | 0.0420 |
