# Supplementary material for: Variation of Soil Microbial Community and Sterilization to Fusarium oxysporum f. sp. niveum Play Roles in Slightly Acidic Electrolyzed Water-Alleviated Watermelon Continuous Cropping Obstacle
Source: Front Microbiol. 2022 Apr 28;13:837121. doi: 10.3389/fmicb.2022.837121 (PMC9097028; doi:10.3389/fmicb.2022.837121)
Supplement: Supplementary Table 2 — The ternary plot of indicator fungi in each treatment of 14 days. Note: the soils with different treatments (Con, Water-7, Water-14, SAEW-7, and SAEW-14) were separately collected from 5 replicated pots for each. Con and control (dry soil); Water-14 (the soil irrigated with deionized water for 14 days); SAEW-14 (the soil irrigated with 60 ppm concentration of slightly acidic electrolyzed water for 14 days). [file Table_2.DOC]

**Supplementary Table 2** The ternary plot of indicator fungi in each treatment of 14 d

| Groups | Enrich | Enrichment | The ratio of  Con (%) | The ratio of  Water-14 (%) | The ratio of  SAEW-14 (%) | p-value | q-value |
| --- | --- | --- | --- | --- | --- | --- | --- |
| Anthophyta | Con | 5.2316 | 78.1176 | 7.2393 | 14.6431 | 0.0087 | 0.0177 |
| Mortierellomycota | Con | 1.5455 | 55.1333 | 14.6809 | 30.1858 | 0.0140 | 0.0223 |
| Chytridiomycota | Con | 0.0982 | 85.0121 | 4.2056 | 10.7823 | 0.0089 | 0.0178 |
| Chlorophyta | Water-14 | 29.4846 | 19.0207 | 63.8492 | 17.1302 | 0.0075 | 0.0178 |
| Ascomycota | SAEW-14 | 87.6113 | 34.7825 | 28.6184 | 36.5991 | 0.0081 | 0.0178 |
