# Supplementary material for: Variation of Soil Microbial Community and Sterilization to Fusarium oxysporum f. sp. niveum Play Roles in Slightly Acidic Electrolyzed Water-Alleviated Watermelon Continuous Cropping Obstacle
Source: Front Microbiol. 2022 Apr 28;13:837121. doi: 10.3389/fmicb.2022.837121 (PMC9097028; doi:10.3389/fmicb.2022.837121)
Supplement: Supplementary Table 3 — The ternary plot of indicator bacteria in each treatment 7 days. Note: the soils with different treatments (Con, Water-7, Water-14, SAEW-7, and SAEW-14) were separately collected from 5 replicated pots for each. Con and control (dry soil); Water-7 (the soil irrigated with deionized water for 7 days); SAEW-7 (the soil irrigated with 60 ppm concentration of slightly acidic electrolyzed water for 7 days). [file Table_3.DOC]

**Supplementary Table 3** The ternary plot of indicator bacteria in each treatment of 7 d

| Groups | Enrich | Enrichment | The ratio of  Con (%) | The ratio of  Water-7 (%) | The ratio of  SAEW-7 (%) | p-value | q-value |
| --- | --- | --- | --- | --- | --- | --- | --- |
| *Actinobacteria* | Con | 24.4670 | 51.3162 | 23.2672 | 25.4166 | 0.0081 | 0.0195 |
| *Chloroflexi* | Con | 18.3797 | 50.6730 | 23.0797 | 26.2473 | 0.0045 | 0.0195 |
| *Gemmatimonadetes* | Water-7 | 13.5745 | 28.3795 | 41.8239 | 29.7967 | 0.0132 | 0.0263 |
| *Acidobacteria* | Water-7 | 9.6515 | 23.6699 | 44.2228 | 32.1074 | 0.0148 | 0.0267 |
| *Bacteroidetes* | Water-7 | 2.4401 | 23.1505 | 38.7370 | 38.1125 | 0.0087 | 0.0195 |
| *Proteobacteria* | SAEW-7 | 21.0850 | 21.4956 | 38.3048 | 40.1997 | 0.0068 | 0.0195 |
| *Armatimonadetes* | SAEW-7 | 0.5667 | 17.5625 | 34.7852 | 47.6523 | 0.0087 | 0.0195 |
| *Nitrospirae* | SAEW-7 | 0.4390 | 13.8202 | 37.4225 | 48.7572 | 0.0060 | 0.0195 |
