# Supplementary material for: Variation of Soil Microbial Community and Sterilization to Fusarium oxysporum f. sp. niveum Play Roles in Slightly Acidic Electrolyzed Water-Alleviated Watermelon Continuous Cropping Obstacle
Source: Front Microbiol. 2022 Apr 28;13:837121. doi: 10.3389/fmicb.2022.837121 (PMC9097028; doi:10.3389/fmicb.2022.837121)
Supplement: Supplementary Table 4 — The ternary plot of indicator bacteria in each treatment of 14 days. Note: the soils with different treatments (Con, Water-7, Water-14, SAEW-7, and SAEW-14) were separately collected from 5 replicated pots for each. Con and control (dry soil); Water-14 (the soil irrigated with deionized water for 14 days); SAEW-14 (the soil irrigated with 60 ppm concentration of slightly acidic electrolyzed water for 14 days). [file Table_4.DOC]

**Supplementary Table 4** The ternary plot of indicator bacteria in each treatment of 14 d

| Groups | Enrich | Enrichment | The ratio of  Con (%) | The ratio of  Water-7 (%) | The ratio of  SAEW-7 (%) | p-value | q-value |
| --- | --- | --- | --- | --- | --- | --- | --- |
| *Actinobacteria* | Con | 24.4670 | 43.4678 | 29.1872 | 27.3450 | 0.0087 | 0.0331 |
| *Chloroflexi* | Con | 18.3797 | 50.2455 | 24.2034 | 25.5510 | 0.0068 | 0.0331 |
| *Proteobacteria* | Water-14 | 21.7036 | 21.2142 | 40.8373 | 37.9485 | 0.0092 | 0.0331 |
| *Patescibacteria* | Water-14 | 1.5277 | 12.7031 | 49.4376 | 37.8594 | 0.0207 | 0.0531 |
| *Cyanobacteria* | Water-14 | 0.3013 | 39.2522 | 46.5875 | 14.1602 | 0.0045 | 0.0331 |
| *Gemmatimonadetes* | SAEW-14 | 11.9207 | 28.4730 | 34.6775 | 36.8494 | 0.0324 | 0.0648 |
| *Acidobacteria* | SAEW-14 | 7.7582 | 26.2743 | 34.2666 | 39.4591 | 0.0070 | 0.0331 |
